# Use of probiotic lactobacilli in the treatment of vaginal infections: *In vitro* and *in vivo* investigations

**DOI:** 10.3389/fcimb.2023.1153894

**Published:** 2023-04-03

**Authors:** Peng Liu, Yune Lu, Rongguo Li, Xiaodi Chen

**Affiliations:** Department of Clinical Laboratory, Jinan Maternity and Child Care Hospital Affiliated to Shandong First Medical University, Jinan, China

**Keywords:** lactobacilli, vaginal microbiome, probiotic, vaginal infections, treatment

## Abstract

The vaginal microbiome is a distinct component of the human microbiome that is colonized by a wide variety of microorganisms. Lactobacilli are the most frequently identified microorganisms in the healthy human vagina. These Gram-positive bacilli can acidify the vaginal microenvironment, inhibit the proliferation of other pathogenic microorganisms, and promote the maintenance of a eubiotic vaginal microbiome. However, a vaginal flora with a reduced proportion or abundance of lactobacilli is associated with various vaginal infections that have been linked to serious health consequences such as infertility, preterm birth, pelvic inflammatory disease, premature rupture of membranes, and miscarriage. Due to their “Generally Recognized as Safe” classification and critical role in vaginal health, probiotic lactobacilli have been widely used as an alternative or adjunct to traditional antibiotic therapy for the treatment of vaginal infections and restoration of the vaginal microbiome. This review focuses on the significant role of probiotic lactobacilli in the vaginal microenvironment and discusses the use of probiotic lactobacilli in the treatment of female vaginal infections *in vitro* and *in vivo*.

## 1 Introduction

The vaginal microbiome is now largely recognized as a balanced ecosystem dominated by *Lactobacillus* species, with notable fluctuation over time and across individuals (Armstrong and Kaul, 2021; Lehtoranta et al., 2022). Under physiological conditions, this *Lactobacillus*-dominant arrangement has long been regarded as an indicator of vaginal health (Cappello et al., 2023; Shen et al., 2023). The production of lactic acid, biosurfactants, bacteriocin-like chemicals, and hydrogen peroxide (H_2_O_2_) by these Gram-positive bacilli, which varies from strain to strain, can acidify the vaginal microenvironment, inhibit the proliferation of other pathogenic microorganisms, and thereby promote the maintenance of a eubiotic vaginal microbiome (Chee et al., 2020; Das et al., 2023). However, a vaginal flora with a reduced proportion or abundance of lactobacilli can lead to vaginal dysbiosis, which is related to vaginal infections such as bacterial vaginosis (BV), vulvovaginal candidiasis (VVC), aerobic vaginitis (AV), human papillomavirus (HPV) infection, human immunodeficiency virus (HIV) infection, herpes simplex virus 2 (HSV-2) infection, and other sexually transmitted infections (STIs) including gonorrhea, trichomoniasis, *Chlamydia* infection, *Mycoplasma* infection, and *Ureaplasma* infection, as well as mixed infections of these diseases (Han et al., 2021).

Owing to their “Generally Recognized as Safe” status, probiotic lactobacilli have been used as alternatives and adjuncts to antibiotics, reducing antimicrobial resistance caused by the overuse of antibiotics in the treatment of illnesses (Aslam et al., 2021; Magalhães et al., 2021; Mei and Li, 2022). In addition, the efficacy of lactobacilli as a prophylactic agent has been demonstrated in long-term administration, although most probiotics are recommended for gastrointestinal use, several are marketed for vaginal health (Lagenaur et al., 2021; Nader-Macías et al., 2021). In recent years, clinical research and development of lactobacilli for the prevention and treatment of vaginal infections in women has advanced rapidly (Lagenaur et al., 2021; Sadahira et al., 2021; Schenk et al., 2021). This paper focuses on the importance of lactobacilli in the vaginal microenvironment and summarizes the use of probiotic lactobacilli in the treatment of several different types of vaginal infections through *in vitro* and *in vivo* investigations.

## 2 Lactobacilli in the vagina and their crucial function

A landmark study conducted by Ravel et al. revealed that the vaginal microbiome of most reproductive-age women is clustered into five microbial community state types (CSTs), four of which are dominated by *Lactobacillus* (CST I: *L.crispatus*-dominated CST, CST II: *L. gasseri*-dominated CST, CST III: *L. iners*-dominated CST, and CST V: *L. jensenii*-dominated CST), where CST IV is characterized by a lower level of *Lactobacillus* and a higher level of anaerobic bacteria (Ravel et al., 2011). Subsequently, Gajer et al. further divided CST IV into two subgroups, CST IV-A and CST IV-B. CST IV-A contains a modest fraction of *L. iners* as well as anaerobic bacteria, where CST IV-B contains a significant amount of BVAB (Gajer et al., 2012). It is worth noting that vaginal CSTs are changeable owing to menstruation, pregnancy, and sexual activity (Amin et al., 2023). For example, *Gardnerella* and *Ureaplasma* are more abundant during the third trimester of a normal pregnancy than during the first and second trimesters, but this change corresponds with a high *Lactobacillus* abundance (Park et al., 2022; Amin et al., 2023).

Lactobacilli can restore the vaginal microbiome to homeostasis *via* several mechanisms. Co-aggregation with pathogens may occur when the cell surface of the lactobacilli contains various mucin-binding proteins, fbronectin-binding proteins, and collagen-binding proteins, and these surface proteins can enhance the ability of lactobacilli to adhere to pathogens (Spacova et al., 2020; Dell'Anno et al., 2021; Zawistowska-Rojek et al., 2022). Production of defense metabolites including lactic acid, H_2_O_2_, bacteriocin, and biosurfactants may also occur. As fundamental components of vaginal defense, D- and L-lactic acid isomers can maintain the pH of the vaginal environment between 3.5 and 4.5, thereby making it inappropriate for the growth of pathogenic bacteria species (Kerry-Barnard et al., 2022). Most lactobacilli produce both the D- and L-chiral isomers of lactic acid. However, *L. iners* can only produce L-isomers. It should be noted that D-lactic acid directly affects host tissues by modulating the immune system and gene expression (France et al., 2022). H_2_O_2_ is an oxidizing chemical that is hazardous to catalase-negative bacteria, including most anaerobes. It exhibits significant antimicrobial action *in vitro*, and colonization with lactobacilli that generate H_2_O_2_ has been linked to a decreased incidence of BV, preterm delivery, and HIV infection (Bonneville et al., 2021). Bacteriocins are a category of potent, ribosomally produced peptides that are active against Gram-positive and Gram-negative bacteria, as well as some fungi (Mokoena et al., 2021; Darbandi et al., 2022). Biosurfactants are a structurally diverse group of surface-active molecules. They can be extremely important in lowering the ability of pathogens to attach to host cells, which is a prerequisite for the growth of biofilms (Nelson et al., 2020; Jeyanathan et al., 2021; Patel et al., 2021). Another mechanism involves competitive adhesion. Lactobacilli adhesins can cause significant adhesion to the vaginal wall. The strong adhesion of lactobacilli to the vaginal epithelium results in exclusion and rejection of harmful pathogens (Lee et al., 2021; Kawahara et al., 2022). Lactobacilli also exhibit immunomodulatory effects by increasing monocytes and macrophages which play a crucial role in the innate immune response *via* activation of Toll-like receptors (TLR) and the generation of cytokines (Mitchell et al., 2021). Specifically, lactobacilli and their derivatives can inhibit the expression of various pro-inflammatory cytokines, such as interleukin (IL)-6, IL-1β, IL-2, and tumor necrosis factor (TNF)-α, and promote the production of IL-10, which can prevent systemic and local acute inflammation (Hao et al., 2021; Hu et al., 2022a). Lactobacilli can also affect the maintenance of epithelial cell tight connections. Lactobacilli can accelerate the re-epithelialization of vaginal epithelial cells and increase the production of vascular endothelial growth factor, an essential factor in tissue healing which is recognized as an epithelial cell migration inducer (Albuquerque-Souza et al., 2021).

As mentioned above, lactobacilli in the vagina are thought to be a key defensive mechanism against infection. Consequently, lactobacilli replenishment of the vaginal microbiome is an intriguing strategy for vaginal infection prevention (van de Wijgert and Verwijs, 2020; Mei and Li, 2022). Furthermore, probiotic lactobacilli have been found to be effective in changing the vaginal microbiota and improving individual health (López-Moreno and Aguilera, 2021). There are approximately 170 species of lactobacilli, but only a few such as *L. acidophilus*, *L. brevis*, *L. crispatus*, *L. delbrueckii*, *L. fermentum*, *L. gasseri*, *L. helveticus*, *L. jensenii*, *L. johnsonii*, *L. plantarum*, *L. paracasei*, *L. reuteri*, *L. rhamnosus*, and *L. salivarius* have been used to treat vaginal infections (Joseph et al., 2021; Piccioni et al., 2021). *L. crispatus* and *L. rhamnosus* are the most often employed species, although *L. rhamnosus* is less prevalent in the natural vaginal microbiome (Veščičík et al., 2020; Petrova et al., 2021). Furthermore, despite the fact that *L. iners* is common in the vaginal environment, it has not been used as a probiotic in the vagina. This may be due to its cultural complexity and uncertain importance (Zheng et al., 2021; Novak et al., 2022).

## 3 Probiotic lactobacilli in the treatment of vaginal infections

**Figure 1:**
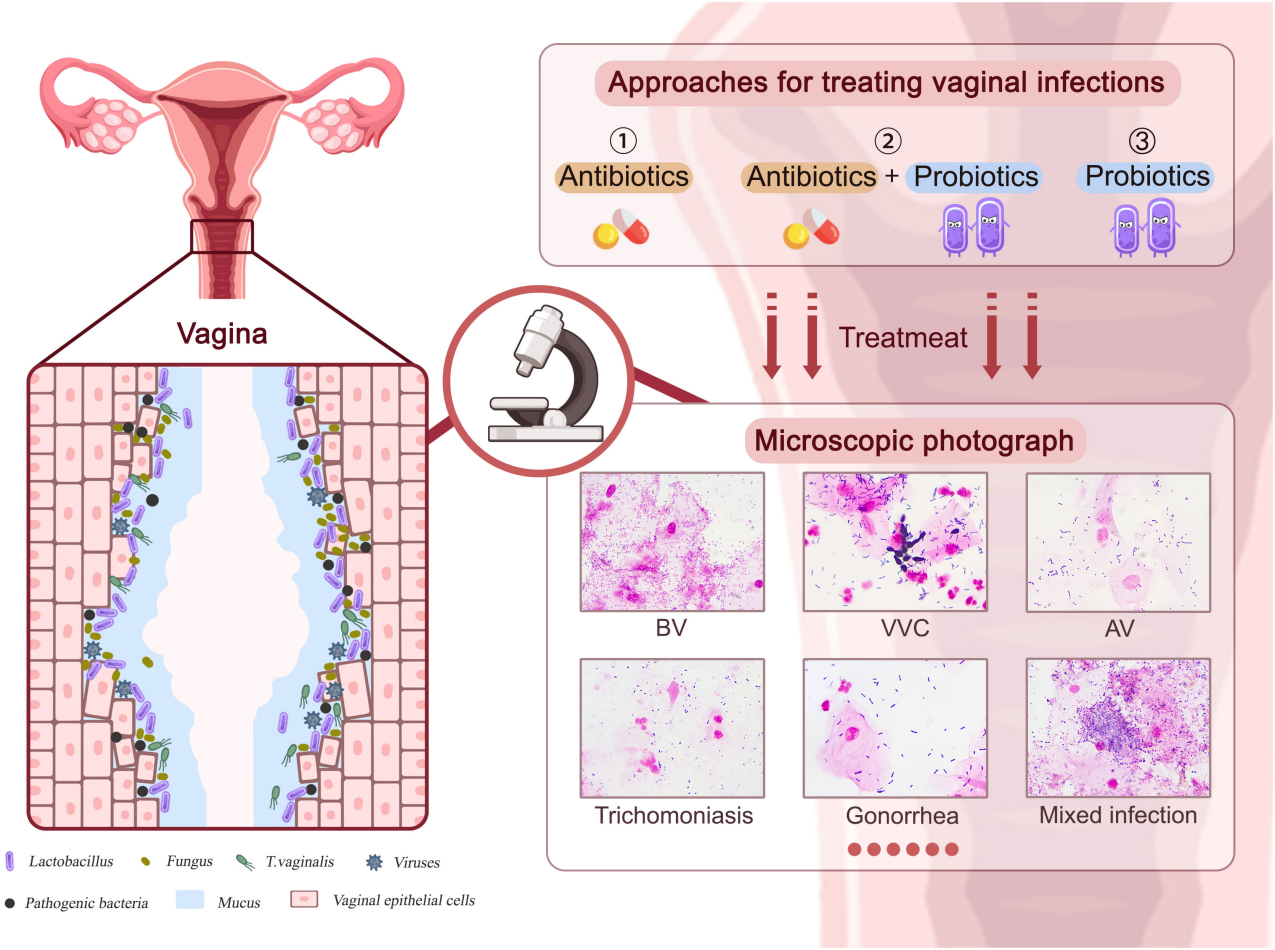
The approaches to treating various vaginal infections. Pathogens associated with BV, VVC, AV, viral infections, and other STIs can cause damage to vaginal epithelial cells and mucosa. Approaches including antibiotics, probiotics, and antibiotics plus probiotics could affect therapeutic efficacy.

Vaginal infections have been linked to serious health consequences such as infertility, preterm birth, pelvic inflammatory disease (PID), premature rupture of membranes, and miscarriage (Huang et al., 2021; Chen et al., 2021a; Dong et al., 2022). As shown in **Figure 1**, vaginal infections such as BV, VVC, AV, viral infections, and other STIs are frequently linked to a vaginal flora containing a lower proportion of lactobacilli and a larger number of pathogens, leading to epithelial cell and mucosal damage in the vagina. The emergence and rapid spread of antibiotic-resistant diseases, particularly multidrug-resistant bacteria, has restricted the use of antibiotics throughout the past century (Larsson and Flach, 2022). Hence, the use of probiotic lactobacilli against pathogens has evolved as an alternative or supplementary vaginal infection therapy. In this chapter, we will outline results from *in vitro* experiments, *in vivo* animal model investigations, and clinical trials of probiotic lactobacilli in the treatment of various vaginal infections.

### 3.1 Probiotic lactobacilli use in BV

BV is one of the most prevalent vaginal diseases in women of reproductive age, and it is characterized by the replacement of beneficial lactobacilli by an anaerobic bacterial overgrowth of *Gardnerella vaginalis*, *Prevotella*, *Atopobium*, and *Mobiluncus* (Chen et al., 2021b; Abou Chacra et al., 2022). Even though multiple studies have demonstrated a link between BV and the presence of a variety of pathogen genera and species, the significance of these organisms in the etiology and pathophysiology of the illness remains unclear (Hu et al., 2022b). In BV therapy, probiotic lactobacilli have been provided either orally or intravaginally for the maintenance and restoration of a healthy vaginal microbiome (Basavaprabhu et al., 2020).

A considerable number of recent *in vitro* investigations have shown that lactobacilli exhibit antibacterial activity against BV pathogens. Several *Lactobacillus* strains isolated from dairy products and the vaginal ecology of healthy women from different countries have antagonistic activity against BV pathogens such as *P. bivia*, *A. vaginae*, and *G. vaginalis* (Happel et al., 2020; Kumherová et al., 2021). More specifically, the acetic acid and lactic acid generated by lactobacilli can alter the morphology of *G. vaginalis* cells, ultimately causing the cells to shrink or burst (Huang et al., 2022). These two chemicals can also impair the activity of the Na^+^/K^+^-ATPase of *G. vaginalis*, resulting in aberrant ATP metabolism and, eventually, inhibiting *G. vaginalis* growth and reproduction (Huang et al., 2022). The cell-free supernatants of lactobacilli have also been shown to dramatically reduce *G. vaginalis* biofilm formation (Qian et al., 2021). Specifically, these probiotics can influence different phases of *Gardnerella* biofilm development and exhibit the highest inhibitory impact when introduced at the early stage of *Gardnerella* biofilm formation (He et al., 2021). Lactobacilli can also prevent the adherence of vaginal infection-causing pathogens such as *Gardnerella* and *Mobiluncus* to vaginal epithelial cells by increasing the production of the proinflammatory cytokines IL-8 and IL-1β, as well as that of human β-defensin 2, and by decreasing the concentration of secretory leukocyte protease inhibitor (He et al., 2020). Quantitative PCR analysis has shown that *L. plantarum* can reduce the pathogenicity of *G.vaginalis* by repressing the expression of the genes related to virulence factors, adhesion, biofilm formation, metabolism, and antimicrobial resistance (Qian et al., 2021).

*In vivo* studies performed on animal models also confirm the benefits of probiotic lactobacilli on BV infection. *L. gasseri* can reduce viable *G. vaginalis* numbers, inhibit sialidase activity, regulate TNF-α and IL-1β expression, and decrease myeloperoxidase activity in experimental mouse models (Zhang et al., 2022). Similarly, in mouse vaginal tissue lysates, a combination of five lactobacilli strains can greatly inhibit *G. vaginalis* proliferation and considerably lower myeloperoxidase activity, pro-inflammatory cytokine levels (TNF-α, IL-1β, and IL-6), and nitric oxide levels (Choi et al., 2022). In experiments using a *Caenorhabditis elegans* model, *L. plantarum* strain P1 has shown potent antibacterial action against *G. vaginalis* and considerably prolonged the lifespan of *C. elegans* after exposure to infection agents (Li et al., 2022).

**Figure 2:**
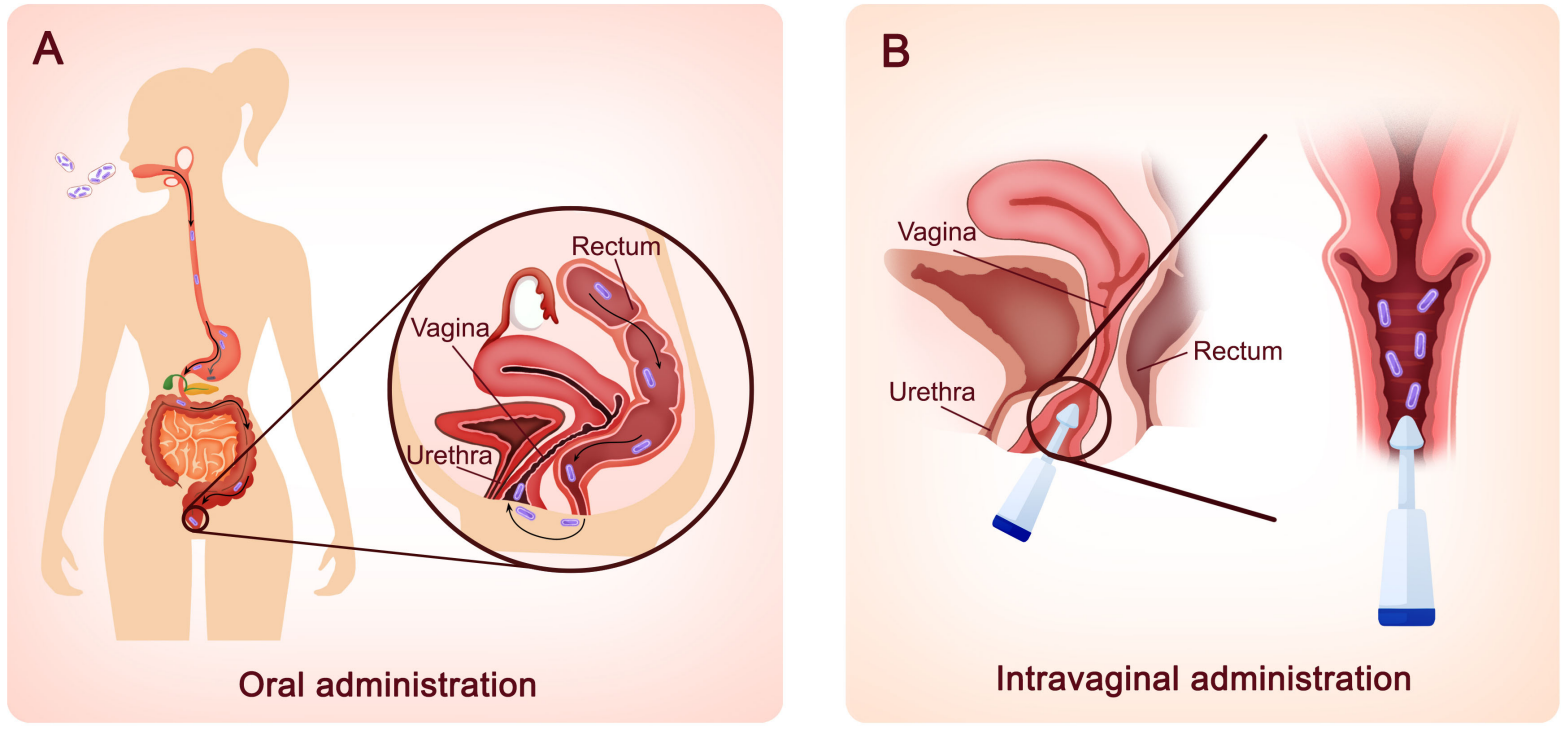
Two different routes for probiotic lactobacilli administration. **(A)** Oral administration. Probiotic lactobacilli taken orally must travel *via* the mouth, stomach, intestines, and colon before reaching the vagina *via* skin contact in the perineum. The probiotics are delivered to the vagina in approximately seven days. **(B)** Intravaginal administration. Probiotic lactobacilli can also be administered intravaginally using external devices. Within 2–3 days, lactobacillus strains manifest their effects.

Based on the positive efficacy of lactobacilli in treating BV infection in animal models, clinical trials on the effectiveness of a single strain or combination of probiotics given orally or intravaginally in the treatment of BV infection have been conducted. Previously, *L. rhamnosus* GR-1 and *L. reuteri* RC-14, the most thoroughly studied vaginal probiotics, have been administered orally to BV patients and have resulted in a notable improvement in vaginal flora (Martinez et al., 2009; Vujic et al., 2013). Recent clinical trials using these two strains, however, have not achieved ideal outcomes. A randomized controlled trial conducted in Shenzhen, China, was performed to evaluate the effectiveness of metronidazole alone and oral probiotics in addition to antibiotics in the treatment of BV. When *L. rhamnosus* GR-1 and *L. reuteri* RC-14 were administered orally for 30 days as an adjunct to metronidazole, no significant difference was seen in the total cure rate (Zhang et al., 2021). One possible cause may be that the *L. rhamnosus* GR-1 and *L. reuteri* RC-14 bacteria isolated from the vaginas of Caucasian and African American women might not work in Chinese people. Therefore, ethnicity might be a key factor. Aside from these two well-known *Lactobacillus* strains, other strains such as *L. acidophilus* GLA-14, *L. crispatus* LMG S-29995, *L. crispatus* DSM32717, *L. crispatus* DSM32720, *L. paracasei* LPC-S01, and *L. rhamnosus* HN001 have also been used orally in clinical investigations (Russo et al., 2019a; Koirala et al., 2020; Reznichenko et al., 2020; Mändar et al., 2023). These trials revealed that oral probiotics can enhance the recovery rate and relieve the symptoms of BV, as well as improve the vaginal microbial profile. As shown in **Figure 2**, in addition to oral delivery, intravaginal administration is becoming increasingly popular among clinical studies (Marcotte et al., 2019; Baldacci et al., 2020; Cohen et al., 2020; Armstrong et al., 2022; Mändar et al., 2023), possibly because intravaginal administration avoids the passage of lactobacilli into the highly acidic stomach, preventing loss of probiotics and accelerating the onset of effect. Compared to orally administered probiotics, which reach the vagina in approximately seven days, *Lactobacillus* strains administered intravaginally show effects in two to three days (Mogha and Prajapati, 2016). Most importantly, fueled by the draft guidance of the Food and Drug Administration (FDA) to develop live biotherapeutic products (LBPs), LACTIN-V (including *L. crispatus* CTV-05) has been developed as an adjuvant therapy and might be the first microbiome-based LBP to prevent the recurrence of BV, a breakthrough in BV treatment (Cohen et al., 2020; Lagenaur et al., 2021; Armstrong et al., 2022).

### 3.2 Probiotic lactobacilli use in VVC

VVC is considered the second most common vaginal infection after BV, and it is mainly caused by the opportunist fungus *Candida* (Willems et al., 2020). According to published research, fluconazole is widely used in the treatment of VVC; however, its function is to inhibit the growth of *Candida* but not eradicate it (Dunaiski et al., 2022). This can lead to the emergence of fluconazole resistance. As an alternative or adjuvant therapy, probiotic lactobacilli have shown considerable promise in the treatment of VVC (Mohankumar et al., 2022; Sun et al., 2023).

*Candida* may undergo a morphological change from round-ovoid yeast cells to hyphal mycelial growing organisms, allowing it to thrive as both a commensal colonizer and a pathogen (McKloud et al., 2021). Studies conducted *in vitro* using vaginal yeast and lactobacilli co-cultures suggest that lactobacilli inhibit the *Candida* yeast-to-hypha switch, reducing the level in the vagina and competing with *Candida* for adhesion sites on epithelial receptors, owning to their higher affinity (McKloud et al., 2021; Spaggiari et al., 2022). For example, lactic acid secreted by lactobacilli can inhibite *Candida* overgrowth and its transition from the commensal yeast form to the pathological hyphal form by modulating *Candida* protein expression, including agglutinin-like sequence protein (AlS3) and the hypha-associated proteins (HGC1, Ece1, Hwp1and Hyr1) (Chow et al., 2021). Furthermore, inhibiting the development of pathogenic hyphal forms of *Candida* species by lactobacilli can restrict the capacity of these yeasts to create biofilms (Nelson et al., 2020; McKloud et al., 2021; Vazquez-Munoz et al., 2022). The biosurfactants extracted from lactobacilli such as iturin, lichenysin, and surfactin have the capacity to limit *Candida* biofilm formation and prevent biomass expansion (Nelson et al., 2020). Lactobacilli can also reduce *C. albicans* pathogenicity by boosting the local immune system response of human cells by modifying immune cytokines (Andrade et al., 2021; Charlet et al., 2022). In particular, the oleic acid and palmitic acid generated by *L. johnsonii* can boost the expression of IL-10 and reduce the production of TNF-α, IL-6, and IL-12 in lipopolysaccharide-stimulated macrophages (Charlet et al., 2022). It should be noted that not all *Lactobacillus* species are advantageous and protective in VVC patients (Kalia et al., 2020). As previously documented, VVC-positive women exhibited an increase in the relative abundance of *L. iners*. *L. iners* can induce a marked increase in biofilm formation by *C. albicans*, enhance hyphal growth, and upregulate expression of the typical hyphae-associated genes *hwp1* and *ece1* (Sabbatini et al., 2021). This might limit *L. iners* use in treating vaginal infections.

Recent investigations in animal models have demonstrated the efficacy of oral and intravaginal probiotic therapy in treating VVC. In a mouse experimental model, biosurfactants derived from a vaginal *L. crispatus* strain can lower viable cell counts of *C. albicans* and leukocyte influx (De Gregorio et al., 2020). Similarly, in the murine VVC model, prophylactic *L. casei*/pPG612.1-BLF treatment increased vaginal mucosa IL-17 production, decreased IL-23 levels, and led to reduced *Candida* burden after 5 days of therapy (Liao et al., 2019).

Notably, several clinical investigations have demonstrated an improvement in the treatment of VVC with or without antibiotic therapy, plus oral or intravaginal probiotic lactobacilli administration. In a randomized, double-blind, placebo-controlled study, eight weeks of probiotic lactobacilli treatment was found to be helpful for pregnant women, particularly in its ability to relieve vulvovaginal symptoms and VVC recurrences, along with decreased emotional and social discomfort (Ang et al., 2022). Similarly, oral or vaginal administration of three *L. crispatus* strains can lower the combined scores of two of the most important symptoms in VVC patients, the amount of discharge and the level of itching/irritation (Mändar et al., 2023). In addition to lactobacilli alone, the combination of lactobacilli with antibiotics is also an effective therapy for vaginal *Candida* infection. By improving the composition of vaginal flora and reestablishing vaginal microecology, probiotic lactobacilli vaginal capsules combined with clotrimazole vaginal tablets can enhance the effectiveness of treatments for simple VVC (Zeng et al., 2023). The combination of *L. acidophilus* GLA-14, *L. rhamnosus* HN001, and bovine lactoferrin dramatically improved itching and discharge in VVC patients at 3 and 6 months, and throughout the six-month follow-up, the intervention group had considerably fewer recurrences than the placebo group (Russo et al., 2019b).

### 3.3 Probiotic lactobacilli use in AV

AV is characterized by a sharply reduced lactobacilli level, high levels of aerobic bacteria, including *Streptococcus agalactiae*, *Staphylococcus aureus*, *Escherichia coli*, and *Enterococcus faecalis*, and vaginal inflammation (Prasad et al., 2021; Wójkowska-Mach et al., 2021). Antibiotics that target aerobic bacteria such as clindamycin, meclocycline, and kanamycin have been used to treat AV (Ma et al., 2022). However, it is highly improbable that oral use of any of the antibiotics listed above have a long-term beneficial effect on the vaginal environment. Regular and sustained use of probiotic lactobacilli is a safer method in women with AV-associated vaginal flora (Das et al., 2023).

The potential benefits of probiotic lactobacilli have been Investigated *in vitro* with AV pathogens. Extracellular vesicles, phenyl-lactic acid, bacteriocins, and exopolysaccharides, isolated from lactobacilli display remarkable antioxidant and antiproliferative activity and inhibit *S. aureus, E. faecalis, S. agalactiae*, and *E. coli* with noticeable antibiofilm activity (Xiu et al., 2020; Abdul-Rahim et al., 2021; Croatti et al., 2022; Jiang et al., 2022). Studies have shown that lactobacilli can inhibit *S. aureus* through interfering with the staphylococcal quorum-sensing system *agr*, a key regulator of virulence genes, and by suppressing the generation of toxic shock syndrome toxin-1 in *S. aureus* (Li et al., 2011; Singh et al., 2022). Most importantly, as the most prevalent risk pathogen for both early-onset and late-onset newborn sepsis, *S. agalactiae* viability can also be inhibited by lactobacilli *via* multiple strategies. Recent studies have shown that supernatants from lactobacilli can inhibit *S. agalactiae* biofilm formation on endometrial cells (Shiroda et al., 2020; Cajulao and Chen, 2021). Lactobacilli can also lessen the pathogenicity of *S. agalactiae* by enhancing the local immunological response of human cells. For example, in a model of *S. agalactiae*-infected primary endometrial epithelial cells, the colonization of lactobacilli can decrease the expression of IL-6, which is elevated in the presence of pathogens, and increase the production of IL-1 (Chenoll et al., 2019).

The potential effectiveness of lactobacilli against AV-associated bacteria has been proven in several studies using animal models. Intravaginally administered *L. reuteri* have reduced physiological index, vaginal bacterial burden, and histopathological changes in vaginal tissues in *E. faecalis* infected mice. The probiotic *L. reuteri* can increase the expression of the anti-inflammatory cytokines Foxp3, interferon-gamma (IFN-γ) and reduce overexpression of the pro-inflammatory cytokines IL-6 and IL-1β caused by *E. faecalis* infection (Shazadi et al., 2021; Shazadi et al., 2022). Similarly, lactobacilli also have a protective effect against *E. coli* infection. By the induction of a host IFN-1 response, which in turn increases the production of cathepsin D within lysosomes to kill the pathogen, *L. crispatus* can alleviate bladder uropathogenic *E. coli* infection in a mouse model (Song et al., 2022). In mice, serial vaginal inoculation with probiotic *L. reuteri* gives partial protection against *S. agalactiae* infections, and this effect is mediated in part by mucosal immunity (Brokaw et al., 2021).

Clinical trials performed to determine whether probiotic lactobacilli can be used to treat AV have demonstrated considerable promise. In a pilot study, pregnant women ingested *L. salivarius* CECT 9145 daily from week 26 to week 38, and the treatment can reduce the number of *S. agalactiae*-positive women throughout pregnancy and the number of women with intrapartum antibiotic prophylaxis during birth (Martín et al., 2019). Similarly, in China, vaginal and rectal *S. agalactiae*-positive pregnant women who consumed two probiotic capsules containing *L. rhamnosus* and *L. reuteri* also significantly decreased the species abundance of *Enterococcus*, *Staphylococcus*, and *Streptococcus* in the vaginal flora and improved pregnancy outcomes (Liu et al., 2020). A meta-analysis also found that taking probiotics throughout pregnancy was related to lower GBS recto-vaginal colonization at 35-37 weeks and a healthy perinatal profile (Menichini et al., 2022).

### 3.4 Probiotic lactobacilli use in vaginal virus infections

HIV, HPV, and HSV-2 are the three most prevalent sexually transmitted viruses that have a substantial impact on female health. According to the statistics provided by UNAIDS for 2018, there were 36.9 million individuals living with HIV and 1.8 million newly diagnosed cases in 2018 (Baxi et al., 2020). More importantly, young women of reproductive age continue to be one of the most vulnerable and impacted populations, with much higher rates of HIV infection than men of the same age (De Oliveira et al., 2017). HPV can infect the stratified squamous epithelium and stimulate cellular proliferation, resulting in benign hyperplasia or in some cases, cervical cancer following prolonged, unresolved infection (Szymonowicz and Chen, 2020; Alimena et al., 2022). HSV-2 can produce genital herpes in the form of painful sores at the site of viral replication and shedding and has the capacity to penetrate the central nervous system and induce latent dorsal root ganglia infection when in touch with the genital mucosal surface (Chentoufi et al., 2022). Because each of these illnesses raises the chance of developing another STI, the epidemiology of these infections is exceedingly convoluted (Torcia, 2019).

Based on its essential role in the female vagina, lactobacilli have shown potential to relieve various viral infections *in vitro*. By blocking HIV-1-cell receptor connections, extracellular vesicles generated by lactobacilli can protect human cervico-vaginal cells. This inhibition was related to a decrease in viral envelope protein exposure due to the steric hindrance of gp120 (Costantini et al., 2022). In addition to wild-type strains, bioengineered lactobacilli that express anti-HIV molecules, such as human CD4, bn nanobodies, have also demonstrated potent anti-HIV activity and have the potential to be used as a live virucide in the vaginal mucosa of women at high risk for HIV infection (Wei et al., 2019; Kalusche et al., 2020). Lactobacilli also possess antiviral activity against HSV-2, and a vaginal microbiome that is dominated by lactobacilli can produce a considerable decrease in the load of HSV-2 in vaginal epithelial cells (Amerson-Brown et al., 2019). HPV is the most common virus among women, and it was discovered that supernatants of lysates and heat-inactivated lactobacilli can suppress the production of the human papillomavirus genes e6/e7, which are the leading cause of cervical cancer (Hu et al., 2021). Additionally, immune-related pathways in cell models indicate that the *L. casei* strain can cause the production of large quantities of IL-2, a cytokine with well-established anti-cervical cancer effects (Jacouton et al., 2019a).

*In vivo* studies in animal models indicate that lactobacilli also possess anti-viral properties. In a humanized mouse model, scientists have engineered *Lactobacillus acidophilus* to express the HIV-1 receptor human CD4 on the cell surface. The modified strains can block HIV-1 infection locally at the intrarectal site of infection but not systemically, reducing the efficiency of HIV-1 sexual transmission (Wei et al., 2019). Similarly, in animal models, lactobacilli have also been modified to express the granulocyte-macrophage colony-stimulating factor and IL-17A cytokine which can cause cytolytic and proliferative responses in splenocytes that are specific to cervical cancer (Jacouton et al., 2019b; Abdolalipour et al., 2022). Therefore, this may be used as a candidate HPV mucosal vaccine with cross-neutralizing activity against diverse HPV types.

Clinical investigations have been conducted to validate the effects of lactobacilli for the treatment of people afflicted with viruses; nevertheless, the findings are not always positive. In a double-blind placebo-controlled clinical trial, intervention with *L. plantarum* and *Pediococcus acidilactici* was found to be safe and to lead to increases in the CD4/CD8 ratio and reductions in sCD14 in HIV-1 infected patients (Blázquez-Bondia et al., 2022). However, using the probiotic *Lactobacillus casei* Shirota had no discernible impact on immunological activation indicators, NK cells, CD4+ and CD8+ subpopulations, or sCD14 levels in HIV-infected patients receiving suppressive antiretroviral treatment with poor CD4+ recovery over the course of a 12-week period (Tenore et al., 2020). Recent preventive HPV vaccinations have been demonstrated to prevent genital infection with multiple HPV types; however, individuals who were infected with HPV prior to vaccination will likely see little benefit. In patients with cervical intraepithelial neoplasia (CIN) 3, an oral agent that expresses the HPV 16 E7 antigen on the surface of *L. casei*, is a breakthrough. Studies showed that 70% of patients receiving the optimized dose encountered a pathological down-grade to CIN 2 after 9 weeks of therapy, and 75% of CIN 3 patients were cured in a subsequent phase 2a clinical trial (Ikeda et al., 2019; Park et al., 2019). Comparable to engineered bacteria, long-term treatment with wild *Lactobacillus* species also demonstrates promising benefits in clinical studies against HPV infection. Long-term use of natural *Lactobacillus* species can change the CST status and increase HPV clearance in women with dysbiosis and concurrent HPV-infections, and thus may have a beneficial influence on HPV infection management (DI Pierro et al., 2021). Acyclovir is the most common first-line therapy in HSV-2 infected individuals with recurrent genital infections (Sharma et al., 2023). A randomized double-blind controlled trial comparing the effectiveness and safety of the multi-strain probiotic and acyclovir found no significant difference in resolution of episode, viral shedding, lesion healing time, or proportion of discomfort, demonstrating the great promise of probiotic lactobacilli against HSV-2 infection (Mohseni et al., 2018).

### 3.5 Probiotic lactobacilli use in other STIs

Apart from the above-mentioned infections, STIs such as gonorrhea, trichomoniasis, *Chlamydia* infection, *Mycoplasma* infection, and *Ureaplasma* infection can invariably result in a major burden for female patients, even though they are not often fatal (Van Gerwen et al., 2022). These STIs are associated with consequences such as PID, ectopic pregnancy, infertility, seronegative arthropathy, and neurological and cardiovascular disorders (Van Gerwen et al., 2022). STIs during pregnancy can also result in fetal or neonatal mortality, early birth, newborn encephalitis, ocular infections, and pneumonia (Glassford et al., 2020). There is an increasing concern for the level of antibiotic resistance in STIs, which is frequently underestimated by clinicians (Tien et al., 2020; Williams et al., 2021). Urgent demand exists for “antibiotic-free” techniques for the control of STIs. Acting as a significant barrier to pathogens in the promotion of female vaginal health, lactobacilli have a great potential for use against vaginal pathogens (Loeper et al., 2018).

Experiments conducted *in vitro* established that lactobacilli can protect the lower female genital tract against infection caused by *N. gonorrheae*. *L. crispatus* can decrease the adhesion and invasiveness of *N. gonorrhoeae* through reducing the expression of genes responsible for pro-inflammatory cytokines like TNF-α and CCL20 in *N. gonorrhoeae*-infected epithelial cells (Płaczkiewicz et al., 2020). Similarly, the cell surface aggregation-promoting factor from *L. gasseri* can block *Trichomonas vaginalis* adhesion to human vaginal ectocervical cells in a dose-dependent manner (Phukan et al., 2018; Malfa et al., 2023). The three main intracellular parasites in the female vagina, *Mycoplasma*, *Chlamydia*, and *Ureaplasma*, have also been shown to be inhibited from infecting the vagina by lactate, bacteriocins, and the acidic environment induced by lactobacilli (Parolin et al., 2018; García-Galán et al., 2020; Garza et al., 2021; Chen et al., 2022). Lactobacilli cells can also directly inactivate the extracellular infectious elementary body of *C. trachomatis* in a concentration-dependent manner, although to a lower extent than their supernatants, by inducing quick and dynamic membrane changes (Parolin et al., 2018).

Several studies indicate that lactobacilli can suppress *Chlamydia* infectivity in animal models, which provides valuable information for the development of lactobacilli as an additional therapy or vaccine for *Chlamydia* infection. Female BALB/c mice intravaginally administered with lactobacilli mixtures, but not a single *Lactobacillus* strain, following genital *Chlamydia* infection showed dramatically reduced levels of *Chlamydia* shedding in both the lower vaginal tract and the intestinal tract, decreased production of cytokines such as TNF-α, IFN-γ, and IL-1β in the vagina, and attenuated upper genital tract pathogenicity and inflammation (Chen et al., 2022). An engineered strain of *L. plantarum* containing the *C. trachomatis* antigen, also known as heterologous immuno-repeat 2, has the potential to be used as a mucosal booster vaccine to elicit H2-specific IgA responses in the vaginal mucosa, which is relevant to the prevention and treatment of *C. trachomatis* genital infections in female B6C3F1 mice (Kuczkowska et al., 2017).

To the best of our knowledge, just one clinical experiment has been conducted evaluating the effectiveness of probiotic lactobacilli against vaginal *Ureaplasma parvum*. Participants in the treatment group of a prospective, monocentric, randomized controlled trial took one sachet a day of a defined probiotic supplement containing four different *Lactobacillus* strains for a period of four weeks. After the intervention period, there was a significant difference in the relative abundance of *U. parvum* between the control group (3.52%) and the intervention group (0.77%) (Schenk et al., 2021).

### 3.6 Probiotic lactobacilli use in mixed vaginal infections

Mixed vaginal infections are characterized by the presence of at least two separate vaginal pathogens at the same time, both of which contribute to an aberrant vaginal environment and result in signs and symptoms (Qi et al., 2021). Even when two different bacteria have been detected in a co-infection, there is still a possibility that one of the infections may not be the source of the vaginal symptoms that are already present (Tumietto et al., 2019). Indeed, it has been documented that the interaction between different pathogens might affect the antibiotic sensitivity of both organisms (Gilbert et al., 2021).

Based on the essential role of lactobacilli in the vaginal microbiome, nearly all probiotic *Lactobacillus* species can combat distinct vaginal pathogens according to *in vitro* studies. Two commercially available *Lactobacillus* strains, *L. acidophilus* GLA-14 and *Lactobacillus rhamnosus* HN001, were tested in a co-culture assays against four distinct pathogens that cause both BV and AV (Bertuccini et al., 2017). *L. rhamnosus* CA15 generated H_2_O_2_, organic acid, and lactic acid, and showed a broad spectrum of antagonistic action and enhanced colonization resistance to urinary tract pathogens, such as *E. faecalis*, *E. coli*, *C. albicans*, *G. vaginalis*, and *S. agalactiae* (Pino et al., 2022a). Similarly, several *Lactobacillus* strains isolated from the vaginal ecology of healthy women also showed antagonistic activity against the pathogens that potentially cause BV, VVC, and AV (Kumherová et al.,2021).

Despite few animal studies have examined the effects of probiotic lactobacilli on mixed infections in the vagina, several clinical trials have examined whether probiotics can enhance the efficacy of antimicrobial treatment for co-infections. The oral probiotic product prOVag^®^, comprised of three *Lactobacillus* strains, decreased the recurrence rate of BV/AV mixed infections by 51% compared with placebo (Heczko et al., 2015). Adding probiotic lactobacilli to the conventional treatment of TV in patients who also had BV can also significantly raise the cure rates of both TV and BV (Sgibnev and Kremleva, 2020). Participants taking daily oral probiotic lactobacilli capsules can lead to a substantial improvement in the vaginal flora in terms of an increase in lactobacilli and a decrease in enterococci, staphylococci, *Gardnerella* spp., and *Candida* spp. in mixed vaginal infected patients (Pino et al., 2022b; Rapisarda et al., 2023).

## 4 Conclusion

Over the past decades, a significant amount of knowledge has been gained regarding the vaginal microbiome and how it is related to host health. A considerable amount of development and advancement have been achieved in the use of probiotic lactobacilli in the prevention and treatment of vaginal infections. The primary benefit of probiotic lactobacilli is the recovery of a healthy, natural microbiome in in the vagina, transforming it from a disease-causing, dysbiotic ecosystem to a healthy, symbiotic microbiome. However, despite the well-established safety of probiotics, not all clinical studies have met their effectiveness objectives. There is a great deal of heterogeneity across clinical trials in terms of probiotic strains/combinations used and the target population by ethnicity, age, life stage, and research methodologies. The FDA has not yet authorized any probiotics for the prevention and treatment of female vaginal infections, thus, these probiotics can only be categorized as safe and beneficial dietary supplements (Stavropoulou and Bezirtzoglou, 2020).

In recent years, studies demonstrating the efficiency of fecal microbiota transplants (FMTs) for the treatment of recurrent *Clostridioides difficile* infections have prompted research into vaginal microbiota transplants as a possible treatment for female vaginal infections (Lev-Sagie et al., 2019). Like FMTs, vaginal samples from healthy donors must be thoroughly tested to rule out infectious microorganisms to prevent severe adverse effects, particularly in immunocompromised patients (Zhang et al., 2018). As opposed to stools, which may be easily obtained from healthy donors, it is difficult to acquire enough vaginal samples from donors due to the small amount of vaginal discharge and the unwillingness of some women to donate. To overcome these restrictions, a platform including transplants of large synthetic microbial consortia, as opposed to individual probiotics or undefined community consortia that may contain infectious pathogens, has been developed. While startup companies like Vedanta Biosciences and the Federation Bio have created artificial microbiome products that are in the commercial development stage, these products are focused more on recurrent *Clostridioides difficile* infection, cancers, inflammatory bowel disease, and allergies (Seydel, 2021; Dsouza et al., 2022). For the treatment of female vaginal infections, it is possible that the synthesis of diverse microbiomes like the CSTs of healthy women will be achieved. Based on the application of emerging sequencing technologies, a synthetic microbial microbiome may have the potential to function as personalized medicine for people who possess complex and distinctive characteristics.

## Author contributions

PL drafted the manuscript for publication. PL and XC prepared the draft of Figures. YL provided the microscope images of **Figure 1**. XC and RL reviewed and revised the manuscript. All authors contributed to the article and approved the submitted version.
